# Primary Squamous Cell Carcinoma of the Thyroid Gland

**Published:** 2018-01

**Authors:** Mohd-Irman-Shah Ibrahim, Yusri-Rahimi Jusoh, Nurul-Nadhihah Adam, Irfan Mohamad

**Affiliations:** 1 *Department of Otorhinolaryngology - Head and Neck Surgery, School of Medical Sciences, Universiti Sains Malaysia Health Campus, 16150 Kota Bharu, Kelantan, Malaysia.*; 2 *Department of Surgery, School of Medical Sciences, Universiti Sains Malaysia Health Campus, Kota Bharu, Kelantan, Malaysia.*; 3 *Department of Pathology, Hospital Sultanah Nur Zahirah, Kuala Terengganu, Terengganu, Malaysia.*

**Keywords:** Squamous cell carcinoma, Thyroid gland, Therapeutics

## Abstract

**Introduction:**

Primary squamous cell carcinoma (SCC) of the thyroid gland is one of the rarest types of all reported thyroid malignancies worldwide. It is very aggressive in nature and carries a poor prognosis. The surgical resection with adjuvant radiotherapy and chemotherapy is the most recommended treatment despite its poor reported outcome.

**Case Report:**

A 74-year-old woman presented with a rapidly progressive neck swelling, with hoarseness and compressive symptoms. Physical examination revealed a multilobulated firm thyroid mass with unilateral vocal cord palsy. Histopathological findings confirmed the diagnosis of SCC while radiological investigations and panendoscopy findings ruled out the possibility of other primary tumors. A surgical intervention was performed; however, the patient eventually succumbed to death prior to undergoing an oncological treatment.

**Conclusion:**

With no standard consensus to guide the management plan, SCC of the thyroid gland presents a great challenge for the managing team to come up with the best treatment option, due to its unfavorable rate of survival.

## Introduction

Primary squamous cell carcinoma (SCC) of the thyroid gland is a rare entity representing less than 1% of all primary carcinomas of the thyroid gland (1-3). Only 84 cases were diagnosed worldwide up until 2012 (3). It is described as a very aggressive tumor with a poor prognosis. The overall survival rate, although poor, is dependent on the extent of the tumor resection and adjuvant radiotherapy/chemotherapy (2,3). 

## Case Report

A 74-year-old woman presented with a painless anterior neck swelling since 2 months. It was progressively increasing in size and was associated with dysphagia, which was non-specific to fluid or solid. A gradual reduction in the appetite and oral intake with worsening dysphagia lead to a significant weight loss within this short period of time. A few weeks prior to the presentation, the patient noticed that her voice started to become hoarse with occasional noisy breathing and shortness of breath. She had no history of neck irradiation or family members with thyroid cancer or any kind of malignancy. During physical examination, she appeared to be cachexic and mildly tachypnoeic with audible biphasic stridor. There was a palpable multilobulated thyroid mass that was hard in consistency. The largest mass was located on the right lobe measuring about 5 cm x 4 cm and extending retrosternally. The trachea was not deviated; however, the normal laryngeal crepitus sign was absent. Nasoendoscopy finding was unremarkable, while laryngoscopy revealed right vocal cord palsy in paramedian position. A panendoscopy was carried out with a negative finding.

**Fig 1:**
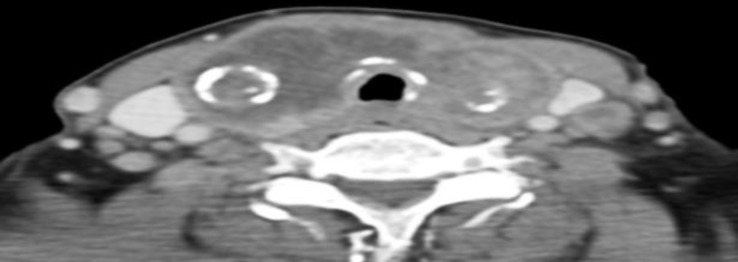
CT showing hypodense mass occupying right thyroid gland, across the isthmus to the left thyroid lobe with a ring calcification feature on right lobe

**Fig 2:**
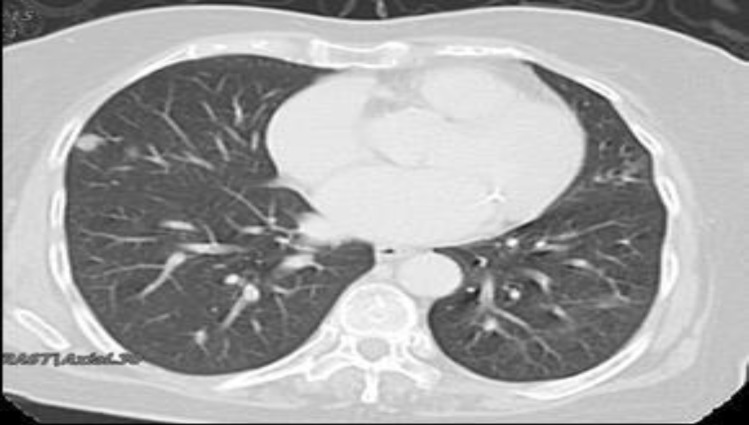
Multiple nodules seen on both lung fields suggestive of bilateral lung metastasis

Fine needle aspiration for cytology (FNAC) revealed a colloid goiter with the presence of malignant cells. Computed tomography (CT) scan revealed the presence of a thyroid mass involving bilateral lobes and isthmus with bilateral cervical lymphadenopathy (Fig.1) with evidence of lung metastases (Fig.2). 

Otherwise, no other features from the imaging could suggest the primary site of the lesion. While the treatment plan was carried out, the patient developed progressively worsening obstructive airway symptoms requiring an emergency tracheostomy. Total thyroidectomy was successfully performed in the following week once her general medical condition had been optimized. Intraoperatively, there were multiple, locally invasive thyroid nodules mainly of the left lobe infiltrating the anterior wall of the trachea into the lumen and laterally to the right lobe with tracheal ring defect. Selective neck dissection was performed to remove the paratracheal and jugulo-omohyoid groups of the lymph node bilaterally. 

**Fig 3:**
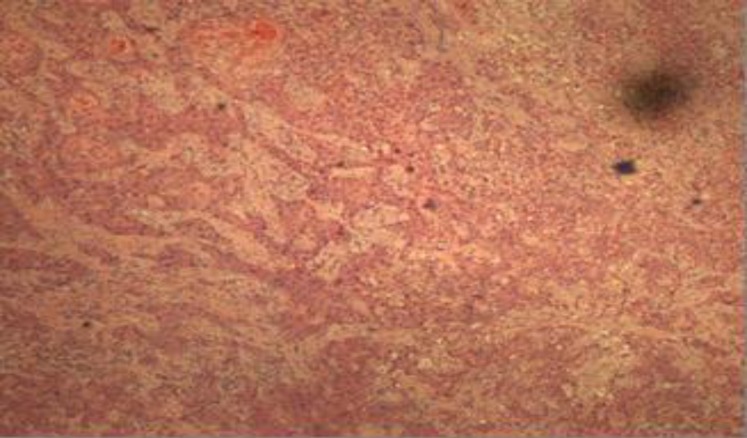
Cohesive malignant cells arranged in sheets, nests, cords, islands and trabeculae pattern [4x, hematoxylin and eosin (HE)]. These malignant cells exhibit large, pleomorphic, hyperchromatic to vesicular nuclei with large prominent eosinophilic nucleoli and abundant eosinophilic cytoplasm

Histopathological analysis of the lesion showed a moderately differentiated squamous cell carcinoma (SCC) whereby the tumor tissue was composed entirely of malignant squamous cells (Fig.3). 

**Fig 4:**
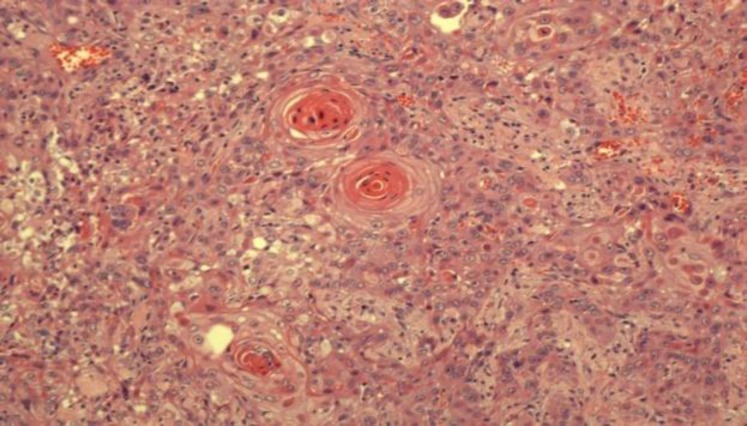
Features of keratin pearls and intercellular bridges. (40x, HE)

These cells were cohesive and arranged in sheets, nests, cords, islands and trabeculae pattern. These malignant cells exhibited large, pleomorphic, hyperchromatic to vesicular nuclei with large, prominent eosinophilic nucleoli and abundant eosinophilic cytoplasm. Individual keratinization and intercellular bridges were noted as well as tumor cell spindling (Fig. 4). Mitotic figures were brisk, including the atypical form, and tumor necrosis was also noted. This tumor was seen infiltrating the surrounding skeletal muscle bundles and destroying the thyroid gland parenchyma.

The patient was closely observed in the intensive care unit (ICU) post operatively and showed good progress. However, 2 weeks post operatively, she contracted a hospital-acquired pneumonia which put her oncological treatment on hold. The infection unfortunately progressed into sepsis and eventually claimed her life a week later. 

## Discussion

Primary SCC of the thyroid gland is an extremely unusual type of thyroid malignancy (1,2,4). It is more common in females, with a mean age of occurrence in the sixth decade (3). Enlarging anterior neck mass is the most common presenting symptom (60%) followed by dyspnea or dysphagia (20%) and change of voice (15%)(3). 

The presence of squamous cells within the thyroid gland has raised question regarding its origin. This leads to the emergence of multiple hypotheses to suggest the etiology and the pathogenesis of how it develops. The “embryonic-rest” theory suggests that these squamous cells could be derived from the remnants of an incomplete descent of the thyroglossal duct cyst, the basaloid cells from the ultimobranchial body, or the thymic epithelium from the third branchial cleft (5-7). Another hypothesis, the “metaplasia” theory, suggests excessive differentiation (metaplasia), which occurs as a result of environmental stimuli such as inflammation (5,6). Moreover, this SCC can also be found in both malignant and benign lesions of the thyroid (5,6), as well as in Hashimoto’s thyroiditis, where it has been shown to have squamous differentiation(6,7). Nevertheless, there were reported cases of pure SCC of the thyroid without other coexisting malignant differentiations identified (6,8). 

SCC of the thyroid gland can either be a primary or secondary disease, in which it could be due to a direct extension of adjacent lesions or metastasis from other primary foci (1,3,4). The latter are 10-times more common(4). However, compared to all thyroid malignancies, the incidence of metastatic thyroid disease is low, at about 2-3%, despite its rich vascular nature(1). Radiological imaging from the neck to the abdomen are important in providing the information to exclude secondary diseases, which can be gathered from either CT or magnetic resonance imaging (MRI). However, there are no specific radiological features to accurately point towards SCC of the thyroid(1). Apart from radiological investigations, the possibility of primary SCC from the upper aerodigestive region, including the pharynx, bronchus and esophagus, can be excluded via panendoscopy (1). The role of FNAC in diagnosing SCC of the thyroid is also very limited(1). More than half of the cases were either reported as papillary carcinoma or non-diagnostic(3). Paradoxically, FNAC results did show high-grade features in 40% of cases (3). With this in mind, high-grade FNAC findings, when combined with clinical and radiological findings, could provide important hints towards primary SCC of the thyroid(3).

Treatment wise, surgical resection of the tumor with adjuvant radiotherapy and chemotherapy is the recommended option (2,7,9). To date, due to the rarity of this disease, there is no standard management outline that can be used as a guideline for its treatment (3,7). The extent of the surgical resection is poorly defined. However, in advanced stage diseases, the extensive and invasive nature of the SCC may prove to be the main factor of surgical failure (3). Moreover, primary SCC of the thyroid is also relatively resistant to radiotherapy(3), while chemotherapy has shown minimal to absent response towards the disease(7). General prognosis of primary SCC of the thyroid is very unfavorable regardless of the treatment, due to its aggressive nature (3,7,9). Cho et al reported a 3-year survival rate of 43.1% of cases where complete resection was performed compared to 15.9% of cases where incomplete resection was performed. 

## Conclusion

Knowing the aggressive nature and the poor outcome of primary SCC of the thyroid, it is best for the managing team to get an early and accurate diagnosis of the disease, which can lead to a more complete tumor eradication thus resulting in a more favorable outcome. However, due to its rarity, the diagnostic conclusion may be challenging and this will further compromise the treatment outcome.
